# Thermal Cloak: Theory, Experiment and Application

**DOI:** 10.3390/ma14247835

**Published:** 2021-12-17

**Authors:** Xiuli Yue, Junyi Nangong, Peiyan Chen, Tiancheng Han

**Affiliations:** 1 National Engineering Research Center of Electromagnetic Radiation Control Materials, University of Electronic Science and Technology of China, Chengdu 610054, China; yue817079@163.com (X.Y.); 2019190603029@std.uestc.edu.cn (J.N.); 2 State Key Laboratory of Electronic Thin Film and Integrated Devices, University of Electronic Science and Technology of China, Chengdu 610054, China; 3 School of Physics and Electronics, Hunan Normal University, Changsha 410081, China; chen622801@icloud.com

**Keywords:** heat transfer, thermal cloaks, transformation theory, scattering cancellation method

## Abstract

In the past two decades, owing to the development of metamaterials and the theoretical tools of transformation optics and the scattering cancellation method, a plethora of unprecedented functional devices, especially invisibility cloaks, have been experimentally demonstrated in various fields, e.g., electromagnetics, acoustics, and thermodynamics. Since the first thermal cloak was theoretically reported in 2008 and experimentally demonstrated in 2012, great progress has been made in both theory and experiment. In this review, we report the recent advances in thermal cloaks, including the theoretical designs, experimental realizations, and potential applications. The three areas are classified according to the different mechanisms of heat transfer, namely, thermal conduction, thermal convection, and thermal radiation. We also provide an outlook toward the challenges and future directions in this fascinating area.

## 1. Introduction

Invisibility cloaks have been popular in the past twenty years with the development of metamaterials [1]. After the realization of invisibility cloaks at microwave frequencies [2], the concept has been extended to various applications including thermal cloaks [3,4,5,6,7,8,9,10], acoustic cloaks [11,12], matter-waves cloaks [13,14], and elastic-waves cloaks [15,16]. The thermal cloak is theoretically designed to make a target invisible under temperature detection [3] or reduce the heat flux in a specific region [7]. These two objectives are expected to be achieved in arbitrary mechanisms of heat transfer, namely, heat conduction, heat convection, or heat radiation. Therefore, we divide thermal cloaks into three sections according to different types of heat transfer. Generally, there are two methods to achieve invisibility: transformation optics (TO) [3,8,17,18,19,20,21,22,23,24,25,26] and the scattering cancellation method (SCM) [4,5,9,10,27,28,29,30,31,32]. The application of TO theory has led to a series of novel optical devices, such as concentrators [33,34], rotators [35,36,37], superlenses [38,39,40], hyperlenses [41,42], artificial black holes [43,44], and bending waveguides [45,46,47]. On the other hand, the SCM has been proposed to design the simple bilayer cloak, based on which a magnetostatic cloak had been created with a ferro-magnetic material and superconductor [48].

Thermal conduction is the primary mechanism of heat transport within solids or among them due to spatial temperature variations. In this situation, TO theory (governed by the wave equation) could be converted into thermodynamics (governed by the heat conduction equation) [4]. Heat is transferred through mass transport in thermal convection. As convection is the chief mechanism of heat transfer in moving fluids, heat and mass transfer are coupled with each other [7]. As a result, the convection–diffusion equation [49,50] and fluid movement [51,52,53,54] need to be considered in thermal convection [7]. The transformation theory has been extended to transient thermal convection in porous media [7]. In thermal radiation, heat is transferred by electromagnetic waves. For manipulating thermal radiation, a structured thermal surface has been reported, which functions as a radiative thermal cloak [17].

In this review, we first introduce the theoretical design of various kinds of thermal cloaks, including conductive thermal cloaks, convective thermal cloaks, and radiative thermal cloaks. Then, we list typical experimental realizations for each kind of thermal cloak and discuss potential applications. Finally, we provide an outlook on the development of this attractive area, as well as challenges to be addressed.

## 2. Conductive Thermal Cloak

### 2.1. TO-Based Thermal Cloak

#### 2.1.1. Theoretical Design

For a TO-based cloak, its thermal conductivity, density, and specific heat capacity can be expressed as [4]:

(1) $${\overset{↔}{\kappa }}^{\prime }=\kappa_{0}\frac{AA^{T}}{\det(A)},\;\rho^{\prime }c^{\prime }=\frac{\rho_{0}c_{0}}{\det(A)}$$

where *κ*_0_, *ρ*_0_, and *c*_0_, respectively, represent the thermal conductivity, density, and specific heat capacity of the background material. $A=\frac{\partial \left(x^{\prime },\;y^{\prime },\;z^{\prime }\right)}{\partial \left(x,\;y,\;z\right)}$ is the Jacobian matrix of the coordinate transformation.

**Figure 1:**
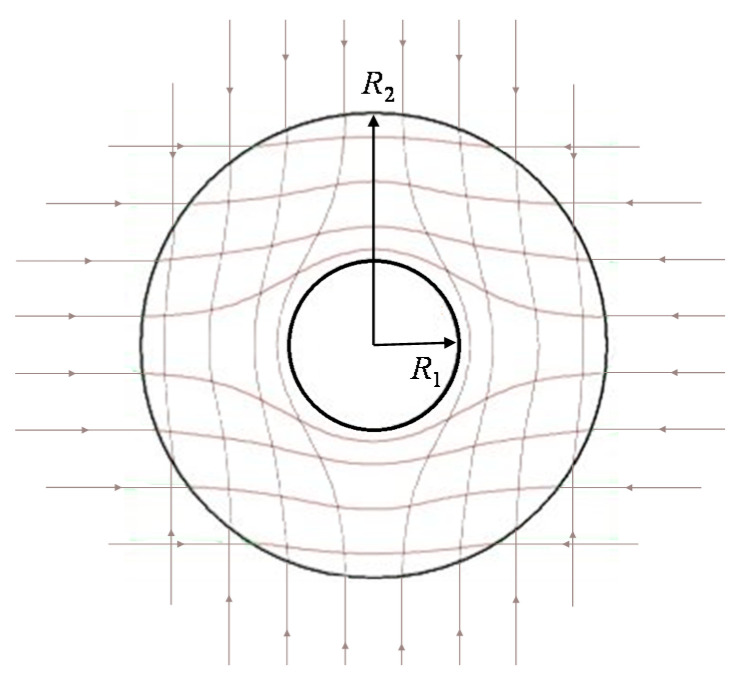
Sketch map of the transformed metric of an invisibility cloak.

In the two-dimensional (2D) case, taking a circular cloak as an example (Figure 1), a circular region (r≤b) in original space (r, φ) is changed into an annular region $\left(a\leq r^{\prime }\leq b\right)$ in a new physical space $\left(r^{\prime },\;\varphi^{\prime }\right)$. Then, we obtain the transformation equation:

(2) $$\{\begin{array}{c} r^{\prime }=a+\frac{b-a}{b}r\\ \varphi^{\prime }=\varphi \end{array}$$

Substituting Equation (2) into Equation (1), we derive:

(3) $${\overset{↔}{\kappa^{\prime }}}_{2D}=\kappa_{0}\left(\begin{array}{cc} \frac{r^{\prime }-a}{r^{\prime }} & 0\\ 0 & \frac{r^{\prime }}{r^{\prime }-a} \end{array}\right)$$

(4) $${\left(\rho^{\prime }c^{\prime }\right)}_{2D}={\left(\frac{b}{b-a}\right)}^{2}\frac{r^{\prime }-a}{r^{\prime }}\left(\rho_{0}c_{0}\right)$$

As an analogy, for a three-dimensional (3D) spherical cloak, the transformation equation can be expressed as:

(5) $$\{\begin{array}{c} r^{\prime }=a+\frac{b-a}{b}r\\ \varphi^{\prime }=\varphi \\ \theta^{\prime }=\theta \end{array}$$

Substituting Equation (5) into Equation (1), we derive:

(6) $${{\overset{↔}{\kappa }}^{\prime }}_{3D}=\kappa_{0}\left(\begin{array}{ccc} \frac{b}{b-a}{\left(\frac{r^{\prime }-a}{r^{\prime }}\right)}^{2} & 0 & 0\\ 0 & \frac{b}{b-a} & 0\\ 0 & 0 & \frac{b}{b-a} \end{array}\right)$$

(7) $${\left(\rho^{\prime }c^{\prime }\right)}_{3D}={\left(\frac{b}{b-a}\right)}^{3}{\left(\frac{r^{\prime }-a}{r^{\prime }}\right)}^{2}\left(\rho_{0}c_{0}\right)$$

Equations (3) and (6) indicate that the transformed conductivities remain inhomogeneous and anisotropic. Meanwhile, the product of density and heat capacity remains inhomogeneous.

In the steady state, the conductivities of a 2D thermal cloak satisfies $\frac{{\kappa^{\prime }}_{r}}{\kappa_{0}}=\frac{\kappa_{0}}{{\kappa^{\prime }}_{\varphi }}=\frac{r^{\prime }-a}{r^{\prime }}$.

It has been proven that the 2D thermal cloak [18] and the 3D thermal cloak [55] can be designed with homogeneous and anisotropic materials, which satisfy:

(8) $$\mathrm{For}\;\mathrm{the}\;2D\;\mathrm{case}:\;\frac{{\kappa^{\prime }}_{r}}{\kappa_{0}}=\frac{\kappa_{0}}{{\kappa^{\prime }}_{\varphi }}=C$$

(9) $$\mathrm{For}\;\mathrm{the}\;3D\;\mathrm{case}:\;\frac{{\kappa^{\prime }}_{r}}{\kappa_{0}}=\frac{\kappa_{0}}{2{\kappa^{\prime }}_{\theta }-1}=\frac{\kappa_{0}}{2{\kappa^{\prime }}_{\varphi }-1}=C$$

where *C* is a constant. Then, a multilayered 2D thermal cloak that consists of two kinds of isotropic materials was experimentally demonstrated [29].

#### 2.1.2. Experimental Realization

The thermal cloak was designed to satisfy the following requirements: (1) to reduce the external heat flux entering the cloaking region to make it colder than its surrounding environment; (2) to ensure that the external temperature distribution is undisturbed. As the ideal transient thermal cloak is difficult to realize in practice according to Equation (3), reduced thermal conductivities and the product of density and heat capacity were mathematically derived in Equations (10) and (11) [4].

(10) $${{\overset{↔}{\kappa }}^{\prime \prime }}_{2D}=\frac{{{\overset{↔}{\kappa }}^{\prime }}_{2D}}{\det\left(A\right)}=\kappa_{0}\left(\begin{array}{cc} {\left(\frac{b}{b-a}\right)}^{2}{\left(\frac{r^{\prime }-a}{r^{\prime }}\right)}^{2} & 0\\ 0 & {\left(\frac{b}{b-a}\right)}^{2} \end{array}\right)$$

(11) $${\left(\rho^{\prime \prime }c^{\prime \prime }\right)}_{2D}={\left(\rho^{\prime }c^{\prime }\right)}_{2D}\cdot \det\left(A\right)=\rho_{0}c_{0}$$

From Equation (10), we can see that only one component of conductivity is spatially varied. More importantly, the product of density and heat capacity is not spatially varied, which removes the obstacle for practical realization.

Schittny et al. [19] performed an experiment of the reduced thermal cloak described in Equation (10). For practical realization, the cloaking shell was discretized uniformly into 5 layers and the conductivity of each layer was approximately homogeneous. To remove the anisotropy, each layer was replaced by two isotropic materials with thermal conductivity $\kappa_{A}$ and $\kappa_{B}$ and thickness $d_{A}$ and $d_{B}$. Then, the multilayered structure was stacked along the radial direction, and the effective thermal conductivities were obtained:

(12) $$\kappa_{r}=\frac{\kappa_{A}\kappa_{B}\left(d_{A}+d_{B}\right)}{d_{A}\kappa_{B}+d_{B}\kappa_{A}},\;\kappa_{\varphi }=\frac{d_{A}\kappa_{A}+d_{B}\kappa_{B}}{d_{A}+d_{B}}$$

**Figure 2:**
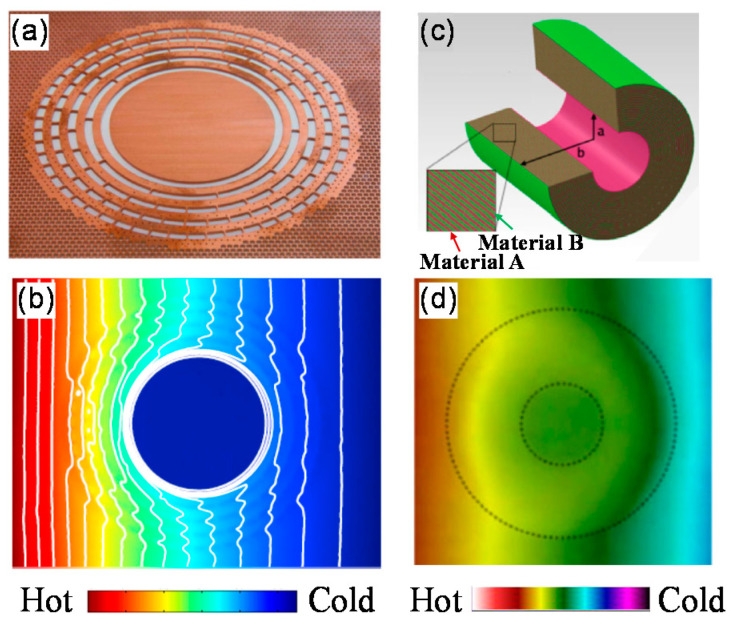
Demonstration of TO-based thermal cloak and multilayered thermal cloak. (**a**) TO-based thermal cloak [19]. (**b**) Measured temperature distribution of (**a**). (**c**) Multilayered thermal cloak [29]. (**d**) Measured temperature distribution of (**c**).

For practical realization, copper and PDMS were used to realize the cloak, as shown in Figure 2a. The measured temperature distribution is shown in Figure 2b. We can see that the central region is colder than its surroundings without disturbing the external temperature distribution.

The realization of a steady-state thermal cloak was first demonstrated by Narayana et al. [29]. A total of 40 alternating layers of natural latex rubber and silicone elastomers containing boron nitride particles were used to construct a multilayered thermal cloak working under agar-water, as illustrated in Figure 2c. The measured temperature distribution is shown in Figure 2d.

### 2.2. SCM-Based Thermal Cloak

#### 2.2.1. Theoretical Design

**Figure 3:**
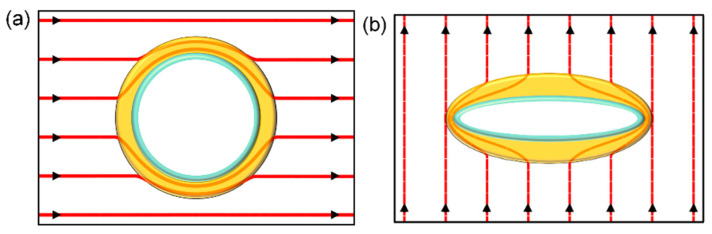
Functional demonstration of bilayer thermal cloak: (**a**) circular shape, (**b**) elliptical shape. The red lines illustrate the heat flux distributions.

Another method to design a thermal cloak is based on the SCM [1]. As shown in Figure 3a, we consider a bilayer thermal cloak at steady state, which comprises an inner layer (a<r<b) and an outer layer (b<a<c) with conductivities of $\kappa_{2}$ and $\kappa_{3}$, respectively. The background conductivity is $\kappa_{0}$. We assume that the inner layer is a perfect insulation material $\left(\kappa_{2}=0\right)$. Without disturbing the external field, we obtain [56]:

(13) $$\mathrm{For}\;\mathrm{the}\;2D\;\mathrm{case}:\;\kappa_{3}=\frac{c^{2}+b^{2}}{c^{2}-b^{2}}\kappa_{0}$$

(14) $$\mathrm{For}\;\mathrm{the}\;3D\;\mathrm{case}:\;\kappa_{3}=\frac{2c^{3}+b^{3}}{2\left(c^{3}-b^{3}\right)}\kappa_{0}$$

From Equations (13) and (14), we can see that the third parameter can be uniquely determined if any two of $\kappa_{0}$, $\kappa_{3}$, and *c*/*b* are known.

We next consider the design of an elliptical bilayer thermal cloak, as shown in Figure 3b [6]. An elliptical cloaking object is wrapped by an insulating layer and a conducting shell. The thermal conductivities of the cloaking object, conducting shell, and background are $\kappa_{1}$, $\kappa_{2}$, and $\kappa_{b}$, respectively. The inner and outer boundaries of the conducting shell are $\xi_{1}$ and $\xi_{2}$, respectively. It is noted that the inner and outer ellipses have the same focus *p*. The relationship between the elliptical coordinate system (ξ, η) and the Cartesian coordinate system (x, y) is written as

(15) $$\{\begin{array}{c} x=p\cosh\xi \cos\eta \\ y=p\sinh\xi \sin\eta \end{array}$$

Then, we consider a uniform heat flux externally applied in the x-direction. The temperature should be close to $-H_{0}p\cosh\xi$ when ξ→∞, where $H_{0}$ is an arbitrary constant. When the external temperature-field distortion is eliminated, we derive

(16) $$\kappa_{b}=\kappa_{2}\coth\xi_{2}\tanh\left(\xi_{2}-\xi_{1}\right)$$

Similarly, when the heat flux is applied in the y-direction, the temperature should be close to $-H_{0}p\sinh\xi$ when ξ→∞. When the external temperature-field distortion is eliminated, we derive

(17) $$\kappa_{b}=\kappa_{2}\tanh\xi_{2}\tanh\left(\xi_{2}-\xi_{1}\right)$$

#### 2.2.2. Experimental Realization

**Figure 4:**
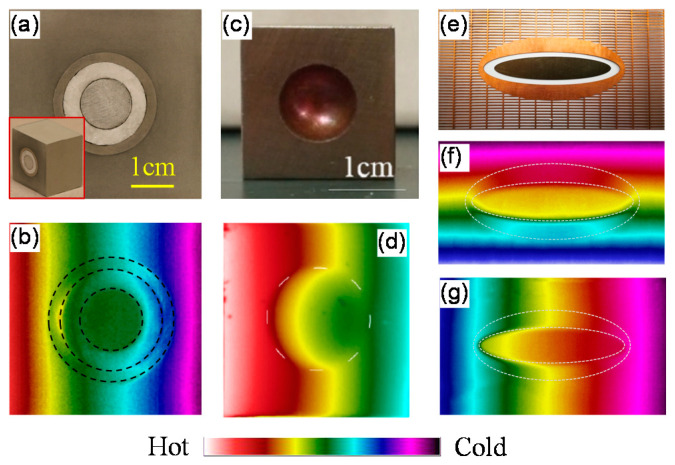
Demonstration of bilayer thermal cloaks. (**a**) Photograph of the 2D cloak [56]; (**b**) measured temperature distribution of (**a**); (**c**) photograph of the 3D cloak [20]; (**d**) measured temperature distribution of (**c**); (**e**) photograph of the elliptical cloak [6]; (**f**) measured thermal profile when heat flows from bottom to top; (**g**) measured thermal profile when heat flows from left to right.

Based on the scattering cancellation method, bilayer thermal cloaks have been demonstrated in both 2D [56] and 3D [20] cases. In the 2D case (Figure 4a), the bilayer cloak is made of expanded polystyrene and Inconel 625 alloy, while the background material is a thermally conductive sealant. The measured thermal profile is shown in Figure 4b. In the 3D case (Figure 4c), the conducting shell is copper and the background is stainless steel. The measured thermal profile is shown in Figure 4d. From the measured results, we can see that the bilayer thermal cloaks function well in the steady-state case. Moreover, these cloaks perform well in the transient state.

The elliptical cloak is illustrated in Figure 4e, in which the shell is copper and an insulating layer is placed between the cloaking target and the shell [6]. The anisotropic background material is achieved with a periodic structure of a T-shaped unit. The required thermal conductivities of the background are satisfied when the period equals 11 mm. Figure 4f,g show the measured thermal profiles when heat flows along the y-direction and x-direction, respectively. We can see that the isothermal lines greatly restore without distortion when the cloak is applied. The elliptical cloak always fulfills its task in the time-dependent case, showing an excellent transient performance [6].

### 2.3. Application

**Figure 5:**
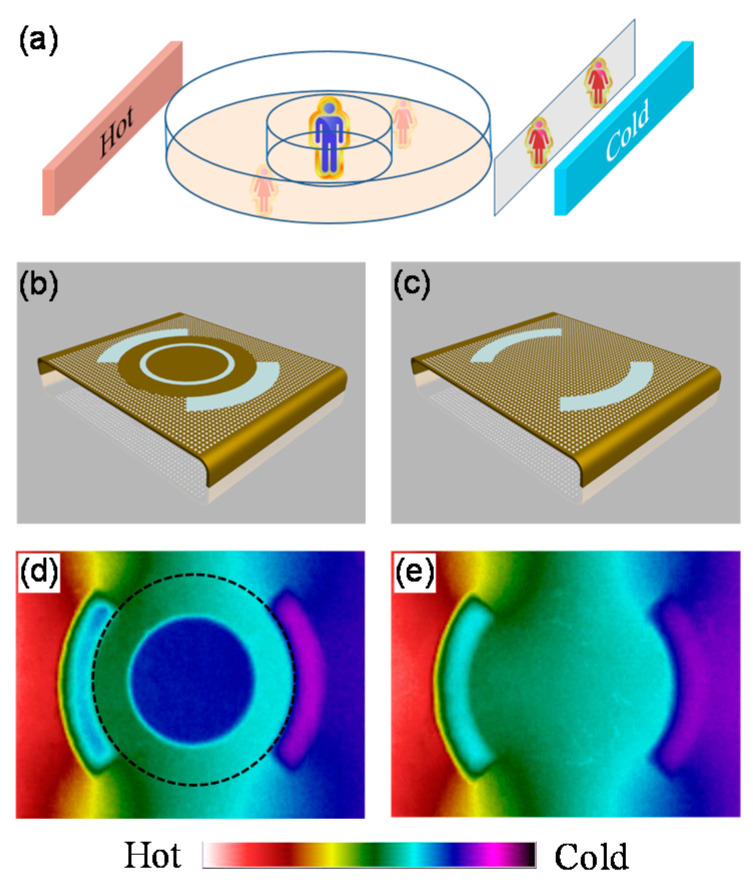
Thermal camouflage [57]. (**a**) Scheme of thermal camouflage; (**b**,**c**) schematic illustrations of the fabricated thermal camouflage and the equivalent object, respectively; (**d**,**e**) measured temperature distributions of (**b**,**c**), respectively.

Based on the thermal cloak, thermal camouflage has been demonstrated, which can change the actual perception into a pre-controlled one [57]. The thermal camouflage device not only makes the object invisible, but also transforms it into other forms. As shown in Figure 5a, the corresponding thermal signature of a “man” (in blue) is the same as that of two “women” (in red) when the “man” is covered by the designed device. Thus, with thermal camouflage, the information obtained from the receiver is false. Figure 5b shows the actual realization of the thermal camouflage device. A thermal cloak is first constructed based on the scattering cancellation method, and then two PDMS wing-ghosts are placed besides the cloak. Figure 5c demonstrates the equivalent situation of Figure 5b. The measured temperature distribution of Figure 5b,c is demonstrated in Figure 5d,e, respectively. We can see that the camouflage device matches very well with that of its equivalent object.

Another application is the macroscopic thermal diode [21]. Inspired by the electronic diode, the thermal diode is capable of conducting heat in one direction while prohibiting heat flow in the opposite direction. To achieve this aim, nonlinear materials are needed. The realization of a macroscopic thermal diode is based on a switchable thermal cloak with different responses to heat flow along different directions. It is worth noting that the switching effect will be triggered when the temperature changes, which is different from the switchable electromagnetic cloaks [58,59,60,61].

**Figure 6:**
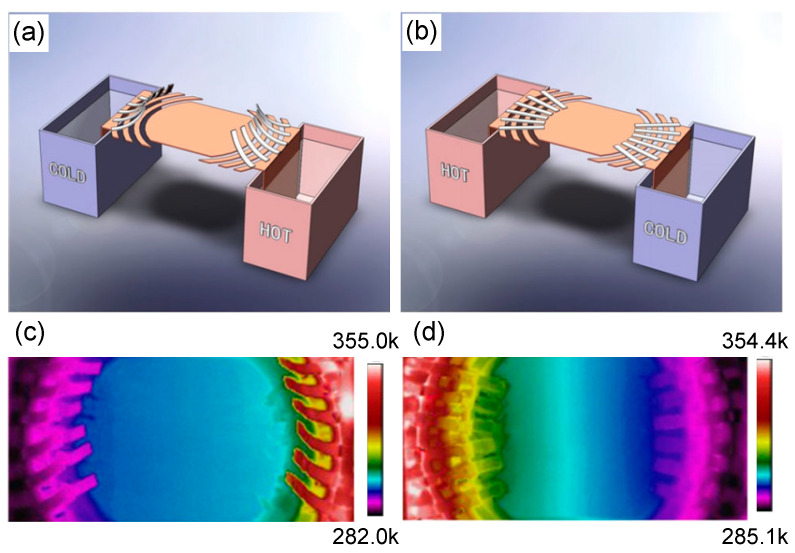
Experimental demonstration of the macroscopic thermal diode [21]. (**a**,**b**) Schematic diagrams of the macroscopic thermal diode in insulation state and conduction state, respectively; (**c**,**d**) measured temperature distributions of (**a**,**b**), respectively.

In practice, the geometrical configuration of devices is supposed to be changed once the temperature varies. The shape-memory alloy (SMA) was used to meet the requirement. The scheme of the macroscopic thermal diode in the insulation state and conduction state are, respectively, shown in Figure 6a,b, where the material of the cloak segments is copper and expanded polystyrene (EPS). When it comes to the critical temperature, the SMA slices will drive the copper slices to connect or disconnect, which brings an abrupt change in thermal conductivity. Figure 6c,d display the temperature distributions of the macroscopic thermal diode in the insulation state and conduction state, respectively. We can see that the temperature distribution in Figure 6c remains almost constant, while an obvious temperature gradient appears in Figure 6d. It is meaningful to realize such a thermal diode as it has great potential related to other methods of thermal manipulation, such as thermal preservation and thermal illusion [57,62].

## 3. Convective Thermal Cloak

### 3.1. Theoretical Design

In convection, heat and mass transfer are coupled with each other [7,63]. In this case, the convection–diffusion equation and fluid movement need to be combined for analysis. The theory for transforming thermal convection at the steady state in porous media has been established based on the convection–diffusion equation [64]. The continuity equation and Darcy’s law are used to form equations, which remain invariant as coordinate systems change [7]. After a series of calculations [65,66,67,68,69,70,71], transformed equations for convection are obtained:

(18) $$\{\begin{array}{c} \vec{v^{\prime }}=-\frac{\beta^{\prime }}{\eta }\nabla p\\ \frac{\partial \left(\phi^{\prime }\rho_{f}\right)}{\partial t}+\nabla \cdot \left(\rho_{f}\vec{v^{\prime }}\right)=0\\ {{\left(\rho C\right)}^{\prime }}_{m}\frac{\partial T}{\partial t}+\rho_{f}C_{f}\left(\vec{v^{\prime }}\cdot \nabla T\right)=\nabla \cdot \left({\kappa^{\prime }}_{m}\nabla T\right) \end{array}$$

where β is the permeability of porous media, η is the dynamic viscosity, p is the intensity of pressure, $\rho_{f}$ is the density of the fluid material, $C_{f}$ is the specific heat of fluid material in porous media, *ϕ* is the porosity, T is the temperature, and $\kappa_{m}$ is the effective heat conductivity calculated using fluids conductivity and solids conductivity $\left(\kappa_{m}=\left(1-\phi \right)\kappa_{s}+\phi \kappa_{f}\right)$.

**Figure 7:**
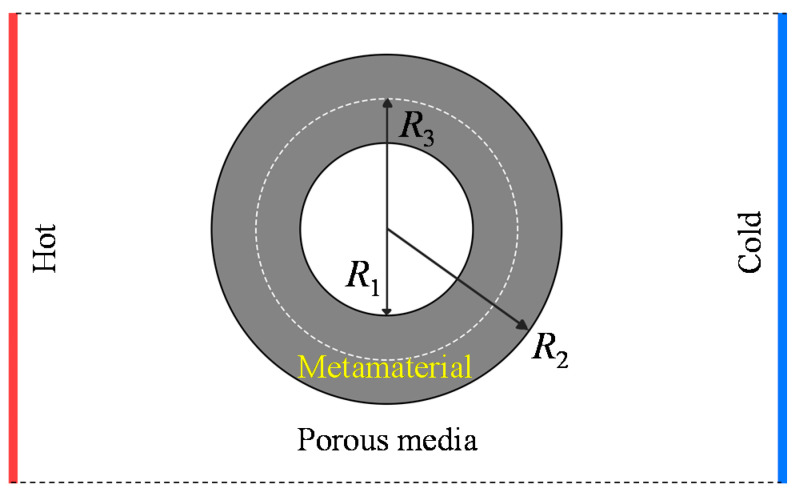
Schematic illustration of a convective thermal cloak.

Figure 7 illustrates the model and the boundary condition under heat convection. Considering a circular cloak, the geometrical transformation can be expressed as

(19) $$\{\begin{array}{c} r^{\prime }=R_{1}+\frac{R_{2}-R_{1}}{R_{2}}r\\ \varphi^{\prime }=\varphi \end{array}$$

### 3.2. Experimental Realization

Typically, mixing natural materials are used to achieve the desired thermal conductivity and specific inhomogeneity. However, its nontunable thermal conductivities and fixed anisotropy lead to great trouble in the adjustment and functional switching of thermal manipulations. In view of this, a tunable analog thermal material was proposed as it could carry out effective conductivity ranging from near-zero to near-infinity [72]. Theoretically, a high effective conductivity can be obtained in the fluid domain through extreme convection [73]. As a result, it is easy to modulate the thermal conductivity by controlling the fluidic rotation speed.

**Figure 8:**
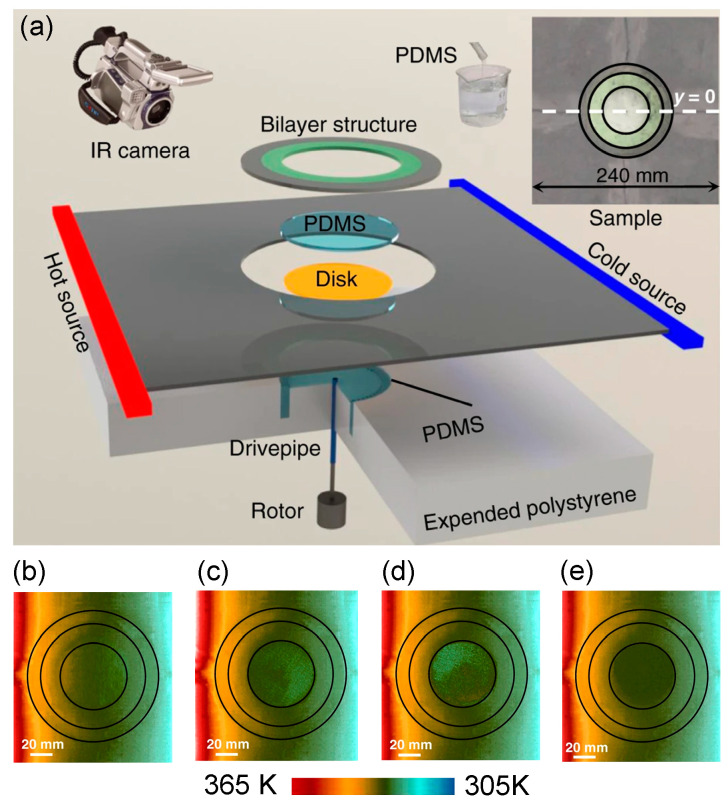
Schematic diagram of the design and measured temperature distribution [72]. (**a**) The experimental setup and the fabricated sample; (**b**–**e**) the measured temperature distributions when the rotation rates are 0 rad/min, 0.6 rad/min, 3.6 rad/min, and 100 rad/min, respectively.

To observe the performance of the convective thermal cloak, an experiment was carried out in Figure 8a [72]. The measured temperature distribution with different rotation rates is demonstrated in Figure 8b–e. From the measured results, we can see that the cloaking performance improves when the fluid rotation rate varies from 0 to 100 rad/min.

### 3.3. Application

One application based on the convective thermal cloak is the thermal meta-device as an analog to zero-index photonics [73]. The zero-index metamaterials [74,75,76] have been used to manipulate thermal emission [77,78], as well as to realize large optical nonlinearity [79] and Dirac cones [80]. Based on the equivalence between the integrated rapid fluid field and a thermal near-zero-index material, a thermal zero-index cloak was experimentally demonstrated [73].

**Figure 9:**
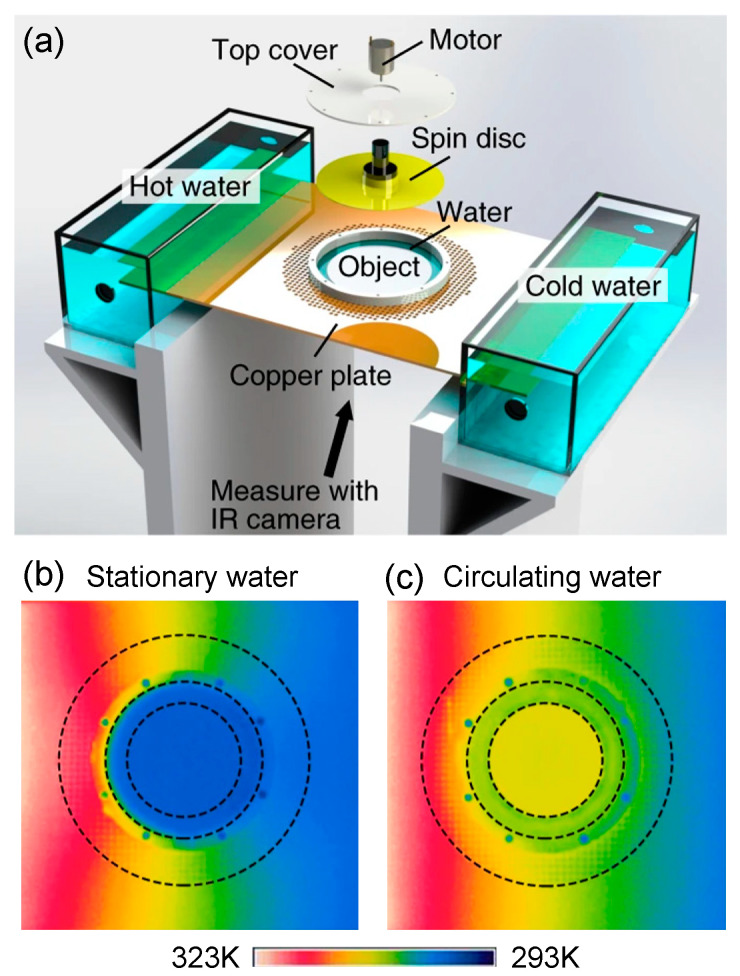
Experimental demonstration of a convective thermal zero-index cloak [73]. (**a**) Experimental setup; (**b**) measured temperature distribution when the water in the central channel is at rest; (**c**) measured temperature distribution when the water is circulating around the object.

The experimental design of thermal zero-index cloaking is illustrated in Figure 9a. In the experimental setup, the water channel surrounding the object is used as the inner layer of the cloak. When the water flows into the channel, it will be driven by a spinning disc attached to an electric motor. The outer layer is built directly through drilling holes to achieve the required conductivity of the background. Figure 9b shows the temperature distribution when the water in the central channel is at rest. Figure 9c shows the temperature distribution when the water is circulating around the object. We can see that the temperature of the central water is colder than its surroundings when the water in the central channel is at rest (Figure 9b). When the water is circulating around the object, a constant temperature is achieved (Figure 9c), which validates the performance of the thermal zero-index cloak.

## 4. Radiative Thermal Cloak

### 4.1. Theoretical Design

A conductive thermal cloak can be achieved based on the scattering cancellation method [81] or transformation theory [82] by tailoring thermal conductivity. However, this methodology does not take effect in the radiative environment, as objects always emit radiative heat in a thermally insulated environment. To get around the bottleneck, Li et al. proposed a new method for the design of a radiative thermal cloak [17].

**Figure 10:**
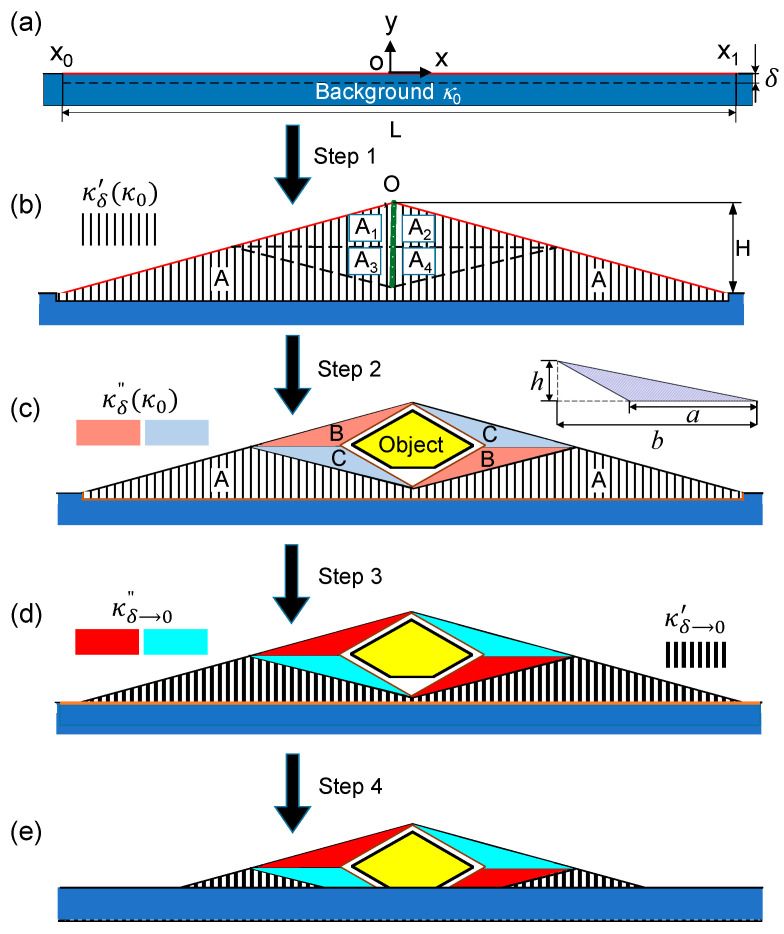
Flow diagram of designing the radiative thermal cloak [17]. (**a**) The background; (**b**) Step 1: space creation; (**c**) Step 2: perform the transformation of a unidirectional thermal cloak; (**d**) Step 3. space elimination; (**e**) Step 4: final design.

A Cartesian coordinate system was built at the center of the background’s upper boundary, as shown in Figure 10a. For the upper boundary, the thermal radiation along the y-direction can be represented as the surface temperature ${T_{0}\left(x\right)|}_{y=0}$. If an object sits on the surface (*y* = 0), the radiation measurement through an IR camera will change due to two reasons. The first reason is that a part of the background is covered by the object, leading to the change in radiation position. We assume that the cover range is from x_0_ to x_1_, and radiation surface is S, leading to a change in surface temperature denoted as ${T\left(x\right)|}_{S}$. The second reason is the change in conduction system due to the introduction of the object, resulting in ${T_{0}\left(x\right)|}_{y=0}\neq {T\left(x\right)|}_{y=0}$. Therefore, the radiative thermal cloak was designed to reduce these two main effects. The following condition is always maintained:

(20) $${T_{0}\left(x\right)|}_{y=0}=\{\begin{array}{c} {T_{0}\left(x\right)|}_{S},x\in \left[x_{0},x_{1}\right]\\ {T\left(x\right)|}_{y=0},x\notin \left[x_{0},x_{1}\right] \end{array}$$

This is not a traditional cloaking problem, as the device is placed directly on the surface of the background. To solve this problem, there are three major steps. The first step is space preparation, where an artificial space is created for operation. Then, a region with width *L* and height δ shown in Figure 10a is considered and the transformation is expressed as follows:

(21) $$x^{\prime }=x;\;y^{\prime }=\frac{\left(\delta +y\right)(L-2\left|x\right|)H}{\delta L}+y$$

which leads to the hatched region in Figure 10b. Based on this transformation, the background heat signature is translated to the upper boundary in Figure 10b. The second step is putting the target object inside the created space using another transformation. A traditional unidirectional cloak [83] is applied along the specific y-direction to force the heat flow direction. The wedge is divided into six parts, as expressed by the dashed lines in Figure 10b, with $h=\frac{H}{2}$ and $b=\frac{L}{4}$. The regions in pink and cerulean are transformed, while the regions labeled “A” remain undeformed, as shown in Figure 10c. The third step is to eliminate the infinitesimal space. As a result, the limit δ→0 is taken to achieve the desired purpose, where the background is not modified, as illustrated in Figure 10d. In step 4, the bottom of the device is truncated to realize contact between the object and the background, as illustrated in Figure 10e. Finally, the thermal radiation from the upper boundary of the device takes the place of that from the object in the y-direction.

### 4.2. Experimental Realization

**Figure 11:**
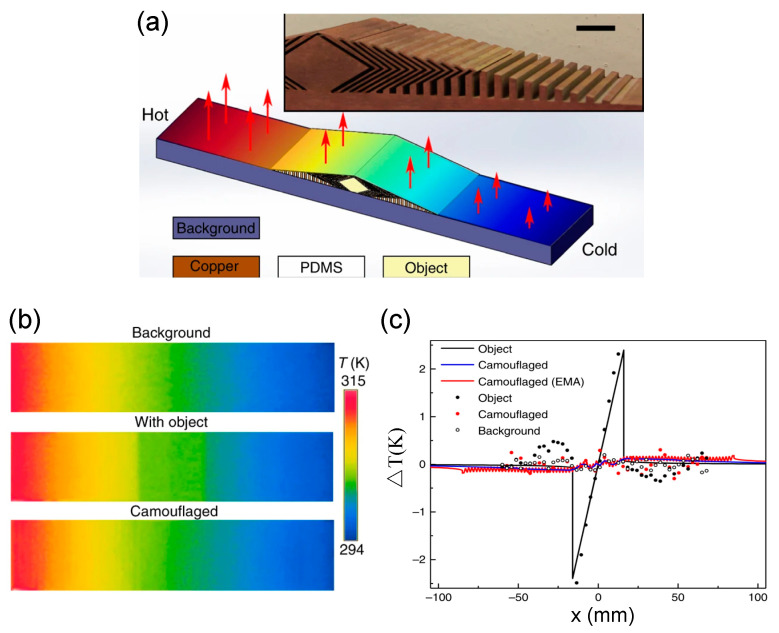
Radiative thermal cloak [17]. (**a**) Photograph of the fabricated sample; (**b**) measured thermal profiles of pure background, with base object, and with radiative thermal cloak; (**c**) quantitative comparison of surface temperature deviation.

The radiative thermal cloak was fabricated with PDMS and a layered structure of copper (Figure 11a) [17]. To examine the performance, the FLIR i60 IR camera was used to observe temperature profiles from the y-direction at the upper surface of the system. The temperature distributions were measured in the three cases: a pure background, a bare object, and the object covered by the cloak. The measured thermal profiles are shown in Figure 11b. Obviously, the object covered by the radiative thermal cloak exhibited the same thermal signature as the background. For quantitative comparison, the temperature variation *T*(*x*) was used to evaluate the impact of the object on the thermal radiation. Then, the surface temperature deviation ΔT(x) was calculated and is plotted in Figure 11c, which validates the design. More recently, the radiative thermal cloak has been extended from two dimensions [17] to three dimensions [84].

### 4.3. Application

Any object with a temperature beyond absolute-zero would emit thermal radiation. Although humans cannot see the infrared radiation, many animals can detect the change in heat energy [85]. Scientists had considered the thermal infrared coating concept as a method to mitigate the emissivity of an object and disturb the thermal signature [86,87], but it is unstable in practice as the camouflage performance is strongly influenced by the environment. Based on a quasi-conformal mapping method, a unidirectional far-infrared cloak that can hide large-scale objects has been experimentally demonstrated [88].

**Figure 12:**
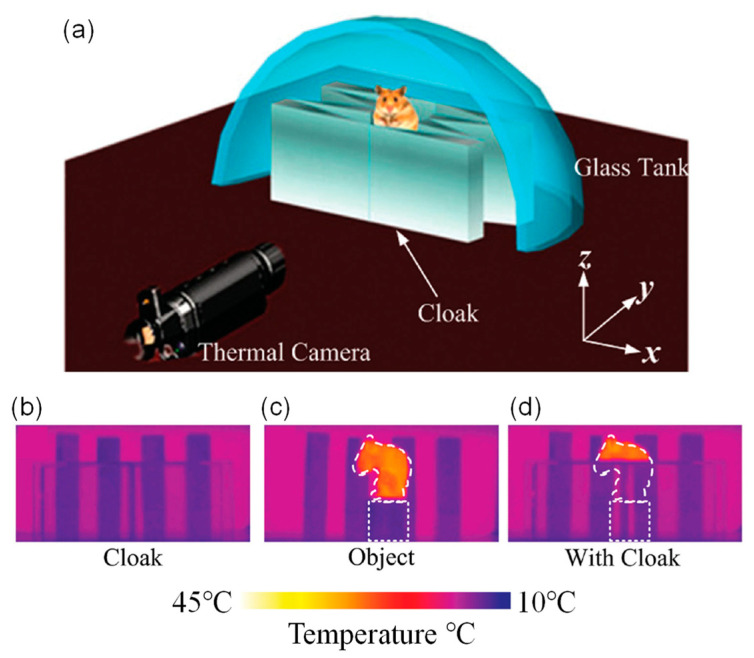
A unidirectional far-infrared cloak for hiding large-scale objects [88]. (**a**) Experimental setup. The thermal images for: (**b**) far-infrared cloak; (**c**) the mouse standing on a cylinder; (**d**) the mouse fixed on a cylinder wrapped by the cloak.

The far-infrared cloak consists of four isosceles triangles and four right-angle triangles. The material is germanium with a refractive index of *n* = 4 [88]. Then, the performance of the far-infrared cloak is examined by hiding a mouse, and the schematic diagram is demonstrated in Figure 12a. In the experiment, a mouse is fixed on the cylinder inside the cloak. For comparison, the head of the mouse is not covered. To adjust flexibly, the background temperature is controlled by placing a glass tank filled with water behind the cloak, where the temperature of water could be changed.

Figure 12b–d show the thermal images obtained by an infrared camera at room temperature (25 °C). We can see that the part of mouse inside the cloak is invisible, while the head of mouse is still visible. Thus, a rat, or a large-scale object, is successfully hidden from thermal detection [88]. Recently, a broadband 3D invisibility cloak made of fast-light media has been reported, which provides a new method for broadband invisibility of large objects [89].

## 5. Conclusions

In this review, we have introduced the recent progress of thermal cloaks for three essential modes of heat transfer, including thermal conduction, thermal convection, and thermal radiation. In the near future, more achievements and breakthroughs can be expected ranging from basic theory [90,91] to potential application [92]. Though great progress has been achieved, a large challenge is still to synergistically use different approaches for more sophisticated and practical heat transfer control [93].

At steady state, both thermal and electric conduction satisfy the Laplace equation, which is promising for the manipulation of thermal-electric fields simultaneously. A bifunctional cloak has been theoretically explored [94] and experimentally demonstrated [95]. In view of the excellent performance of the thermal-electric cloak, it is attractive to achieve a multi-physics cloak that manipulates more than two physical fields in the future.

Janus metamaterials or meta-devices are artificial devices, which are designed to integrate two or more functionalities into one element [96,97,98,99,100]. A path-dependent thermal meta-device has been demonstrated more recently [101]. Meanwhile, new theoretical approaches in thermotics have been proposed to achieve path-dependent meta-devices [102]. Designing a more compact and miniaturized system integrating more functionalities is an active research field.

Beyond transformation theory [3,4] and the scattering cancellation method [103], the topology optimization method has been employed to design thermal metamaterials [104,105]. By using the topology optimization method, arbitrarily irregular-shaped metamaterials can be designed. In addition, a bifunctional cloak for manipulating heat flux and direct current simultaneously has been demonstrated based on topology optimization [106].

## Data Availability

The data presented in this study are available on request from the corresponding author.
